# Delirium researchers’ perspectives of the challenges in delirium biomarker research: A qualitative study

**DOI:** 10.1371/journal.pone.0243254

**Published:** 2021-04-07

**Authors:** Ingrid Amgarth-Duff, Annmarie Hosie, Gideon A. Caplan, Meera Agar

**Affiliations:** 1 IMPACCT (Improving Palliative, Aged and Chronic Care through Clinical Research and Translation), University of Technology Sydney, Sydney, Australia; 2 School of Nursing Sydney, The University of Notre Dame Australia, Sydney, Australia; 3 The Cunningham Centre for Palliative Care Research, St Vincent’s Health Network Sydney, Sydney, Australia; 4 Prince of Wales Clinical School, University of New South Wales, Sydney, Australia; 5 Department of Geriatric Medicine, Prince of Wales Hospital, Sydney, Australia; 6 South West Sydney Clinical School, University of New South Wales, Liverpool, Sydney, Australia; 7 Ingham Institute of Applied Medical Research, Liverpool, Sydney, Australia; University of Iowa Hospitals and Clinics, UNITED STATES

## Abstract

**Background:**

Despite the prevalence and impact of delirium, its pathophysiology remains unclear. In order to advance this field of research, robust scientific methodology is required, yet quality of reporting in this field of research has been highly inconsistent. Delirium biomarker research poses several challenges, none of which have been documented in the literature before. The aim of this study was to explore the perspectives of delirium researchers about key methodological issues in delirium biomarker research.

**Methods:**

Following a Delphi study with delirium experts resulting in 60 recommendations for reporting delirium biomarker studies, semi-structured interviews with international delirium researchers were conducted. Interviews were audio-taped and transcribed verbatim, followed by thematic analysis of the qualitative data.

**Results:**

Fifteen participants were interviewed between August and November 2019. Most were male (n = 12; 75%), clinician researchers (n = 13; 86%), and had more than ten years’ experience in conducting delirium research (n = 9; 60%). Analysis revealed two major themes and ten sub-themes, outlining key considerations to advance the field of delirium biomarker research. The major themes were: 1) Practical and scientific challenges of delirium biomarker research: stagnation versus driving improved methods and reporting; and 2) Valuing delirium research through investment and collaboration.

**Conclusion:**

Findings identified a range of factors that contribute to the practical and ethical challenges of conducting delirium biomarker research, which have not previously been explicitly acknowledged or reported. A clear vision for collaborative efforts to enhance research quality for improved impact was also presented by the delirium researchers. This work complements the preceding Delphi and together these studies provide an in-depth understanding of what is needed in the field to inform and improve methods and reporting of delirium biomarker research.

## Introduction

Delirium is a common, serious and complex neurocognitive condition which is often precipitated by medical illness and hospitalisation [1]. The hallmark features of delirium include changes in attention, awareness and cognition, which variously affect memory, language and visuospatial ability, orientation and perception [2]. Delirium is associated with multiple adverse clinical outcomes including high levels of patient and caregiver distress, significant morbidity and mortality, impairment in activities of daily living, and significant costs to the healthcare system [3–6].

Delirium prevalence in medical in-patients at admission to hospital has been shown to range between 10 and 31%, with incidence of new delirium during admission ranging from 3 to 29% [7]. Occurrence rates for delirium per admission ranged between 11 and 42% [7]. Despite the high prevalence and impact of delirium, knowledge of its pathophysiology is unclear. Current hypotheses include: neuronal ageing, neuroinflammation, oxidative stress, neuroendocrine dysregulation, and disruption to the circadian rhythm [8]. To date, there has been remarkably high heterogeneity of delirium biomarker findings addressing these hypotheses. Other challenges to understanding include unsettled questions about whether delirium represents a single, unified physiological condition or whether there are physiologically discrete subtypes [9]; and ongoing terminological confusion (e.g., delirium vs acute encephalopathy) that drives specialty-specific silos [10]. These high-level issues in the conceptualization of delirium mean that high quality methodological approaches to biomarker research are critical to accelerate understanding of delirium pathophysiology in order to lead to potential therapies.

However, a systematic review of biomarkers in delirium by Amgarth-Duff et al. (2020) [11] highlighted many quality issues in the reporting of delirium biomarker studies. The overall low quality of studies has limited the reliability of outcomes, comparability of results, and ability to synthesise results to develop empirical understanding of delirium pathophysiology. This poor quality reporting has likely contributed to heterogeneity of findings and biological and conceptual uncertainty [12]. In response to the need to improve the field of delirium pathophysiology, a Delphi study was conducted [13] to gather opinions of international experts on delirium research methodology that resulted in a list of reporting guidelines for future delirium biomarker studies. To supplement these recommendations, interviews with Delphi participants and other delirium researchers were then undertaken for an in-depth exploration into the more complex aspects of biomarker study methods and those with a range of methodological options. The consensus and primarily quantitative approach of the Delphi method was not suited to fully explore these aspects; and, furthermore our present goal was not to obtain recommendations but rather to understand the key considerations and the reasons underpinning them. Therefore, the aim of this study was to explore the perspectives of delirium researchers about key methodological issues in delirium biomarker research.

## Methods

### Design

A qualitative study using semi-structured interviews reported in accordance with the Consolidated Criteria for Reporting Qualitative Research (COREQ) [14].

### Participants

Eligible participants were researchers, clinicians and basic scientists with experience in delirium research in either humans or animals, including but not restricted to biomarker research. There was no pre-specified minimum number of years of clinical or research experience; however, experience in delirium research was required to have been in the last ten years to ensure recent knowledge of the study topic.

### Recruitment

Purposive sampling was employed whereby potential participants were actively selected to take part [15]. This was achieved by emailing the international delirium researchers who completed the final round of the Delphi study [13] and other delirium researchers who were not involved in the Delphi process (n = 27) and asking them to participate in a semi-structured interview. Delirium researchers were identified by authorship of relevant papers in the field of delirium, as well as through the lead researchers’ supervisory networks. Snowball sampling [16] was also employed by asking invitees whether they knew any other relevant persons who may be interested in participation. Those who indicated willingness to participate were emailed a participant information sheet and a consent form by the researcher (IAD), which was required to be signed and sent back prior to the interviews taking place. The participant information sheet explained the aim of the study: general content to be discussed, anticipated length of the interview, measures for privacy and confidentiality, and use of data for academic and research purposes.

### Data collection

The interview guide was aligned with the key findings from the earlier Delphi study, while also allowing other topics to arise [13] (Box 1). The interviews were conducted individually, limiting the influence of group bias. The three key areas explored were: 1) the practical challenges of conducting delirium biomarker research, and how they can be overcome; 2) how to account for underlying conditions that are present in many patients with delirium; and 3) the key gaps and methodological shortcomings in current delirium biomarker studies. Questions were open-ended and designed to gain an in-depth understanding of the challenges and nuances of delirium biomarker methodology. The interview guide was piloted with two clinicians who did not formally take part in an interview. The first had extensive experience in delirium research, and the other had clinical experience of caring for patients with delirium. The final interview guide is presented in Box 1.

**Table:** 

**Box 1. Interview guide.**
1. Delirium is a condition that often occurs in the context of other conditions with similar pathophysiological processes. What are your thoughts on accounting for co-existing conditions such as cancer in delirium biomarker studies?
2. Delirium biomarker research poses many practical challenges. In your experience, what some of the key challenges and some ways to overcome these challenges?
3. Where do you think current biomarker studies are falling short?
4. Do you have any comments on the Delphi statements? (for Delphi participants only)
5. Is there anything else you would like to add before we finish up?

All interviews were conducted by the lead author (IAD), a female research assistant and PhD candidate who holds undergraduate and honours qualifications in biomedical science. IAD has prior interviewing and qualitative analysis experience and an in-depth knowledge of existing deficiencies in the quality of reporting of delirium biomarker research [11], but no prior experience of conducting biomarker research. There were no pre-existing relationships between IAD and participants, although the remaining authors knew some of the participants through delirium research collaborations, conferences and advocacy networks. IAD had minimal contact with participants from the time of the Delphi through to the interviews, with the exception of scheduling interviews over email. During telephone interviews, IAD was located in a private office. Data collection continued until no new information emerged (i.e. data saturation). All interviews were audio recorded and transcribed verbatim in a de-identified format.

### Data analysis

A combination of inductive and deductive thematic data analysis [17] was used, as follows:

#### Deductive thematic analysis

Firstly, key areas identified in Round 1 qualitative analysis of the modified Delphi study [13] that were too complex to be resolved through a consensus process (and therefore required a more in-depth analysis) formed the framework for the interview guide. The lead author (IAD) familiarised herself with the data through the transcription process and rereading of the final transcripts. Line-by-line coding of the transcripts was conducted, and a coding tree was developed to elucidate categories. Categories were then collapsed into themes. To ensure rigour, preliminary themes were independently identified by two researches (IAD and AH) and refined collaboratively until the final themes and sub-themes were established.

#### Inductive thematic analysis

Initial data coding was guided by the semi-structured interview questions, with codes and collated data examined for potential sub-themes. Codes were considered important if they were mentioned more than once. IAD identified preliminary sub-themes, that were then refined through an iterative process until the final sub-themes were confirmed by a second researcher (AH).

Data were managed using NVIVO QSR International Pty Ltd. Version 12 software package.

### Trustworthiness of the data

The procedures used in this study were guided by the four general types of trustworthiness in qualitative research, namely: credibility, transferability, dependability and confirmability. Trustworthiness of the data was achieved by using purposive sampling, targeting delirium researchers from a broad range of contexts and countries. The voices of the participants were widely represented in the quotes which supported the themes and achieved transparently in the data interpretation. Discussion among co-authors were also used to enhance the trustworthiness of the data analysis.

### Ethical considerations

Ethical approval for the interviews was obtained from the University of Technology Human Research Ethics Committee on 25/01/2019 (HREC ETH18-2673).

Participant lists were stored on a password protected computer and all participant names were removed from the data transcripts. Participant confidentiality, privacy and anonymity were ensured through the allocation of participant ID codes in the transcripts and manuscript. Data were only accessible to the lead author (IAD) and de-identified data were only shared with the other authors (MA, AH and GC) for their input into analysis and interpretation.

## Findings

Fifteen delirium researchers participated in semi-structured interviews between August and November 2019. Most participants were male (n = 12;75%), clinician/researchers (n = 13;86%), had conducted five or more delirium studies (n = 12;80%) and had more than 10 years’ experience in delirium research (n = 9;60%). Participants were from Europe (n = 7), USA (n = 3), Australia (n = 2), the United Kingdom (UK) (n = 2) and South America (n = 1). Demographic characteristics of participants are outlined in Table 1. Although participants had the option of attending a face-to-face or a telephone interview, all participants opted for a telephone interview. Interview duration ranged from 18–80 minutes (mean 37 (±16)).

**Table 1:** Participant demographics (n = 15).

	n (%)
**Gender**	
Male	12 (80)
Female	3 (20)
**Continent**	
Europe	6 (40)
USA	4 (27)
Australia	2 (13)
UK	2 (13)
South America	1 (7)
**Years in delirium research**	
10+	9 (60)
5–10	3 (20)
1–5	3 (20)
**Current role**	
Clinician/researcher	13 (87)
Researcher	2 (13)
**Number of delirium studies conducted**	
10+	7 (47)
5–10	5 (33)
1–5	3 (20)
**Number of biomarker studies conducted**	
10+	3 (20)
5–10	2 (13)
1–5	5 (33)
0	5 (33)

Thematic analysis resulted in two major themes and ten sub-themes.

1. Practical and scientific challenges of delirium biomarker research: stagnation versus driving improved methods and reporting
  - Accuracy of diagnostic assessment of delirium
  - Delirium superimposed on dementia (DSD)
  - Hypothesis driven
  - Limited infrastructure and resource investment
  - Fluctuating nature of delirium means time point of biomarker collection is a crucial consideration
  - Collecting CSF and imaging in people with delirium
  - Accounting for the complexity/biology of the whole person
  - Standardise delirium biomarker research
2. Valuing delirium research through investment and collaboration:
  - Ethics committee barriers
  - Transdisciplinary collaboration

### Practical and scientific challenges of delirium biomarker research: Stagnation versus driving improved methods and reporting

Participants generally asserted that delirium biomarker research is an extremely difficult and complex field:

*“Yes well the hard thing with this is it is such a complex area and no one actually knows*. *People know what you have to do but they don’t know how to get there*. *It’s very difficult*. *It’s a very grey area*.*” (P09*)

Some expressed a sense of frustration, stagnation and pessimism in the field, due to the complexities, challenges and overall uncertainty:

“*It’s a difficult field*. *There is quite a lot of frustration*. *There are no quick wins*. *There is no money coming into the research*. *I’m not frustrated but I am seeing more difficulties and I am not sure how to get around them in the long run because ethics committees get more difficult*, *money gets scarce*, *the pressure of clinical work […] I’m such a pessimist*! *But that’s the way I see the course of delirium research going in our institution*.*” (P03)*

The need to branch out from siloed investigations and from biomarkers already shown to be associated with delirium was noted:

“*In the 1940’s they found similar things to us now*. *And it’s like… ok let’s move forward*! *[…] I think there is some element of reconfirming*. *But I also think there are some elements of splitting it into medical delirium*, *or ICU delirium–it’s important but we have kind of just got so into that that we have delirium in the cardiac population*, *delirium in the vascular population*, *and delirium in…you know*. *We have so many of these little pocket categories*. *We are reconfirming results because we are interested to see if it’s the same in those populations which is good but I also think it’s kind of not leading to a huge mass of knowledge […] I think it’s time we either need to branch out*, *or use a different method*.*” (P07)*

Delirium biomarker research was perceived to have been a “*hype*” that has since been dulled as there have been no *“quick wins” (P03)*, which ironically had become a short-term enterprise:

“*Delirium is something like a hype*. *Everyone was very excited when the first paper came out–the one from the States*, *but it’s gone a bit quiet since then because I think we all realise it’s not going to be a quick win*. *So we try to focus on something that is easy to sell*.*” (P03)*

#### 1a. Accuracy of diagnostic assessment of delirium

Participants perceived clinical recognition of delirium to be generally poor, adding to the difficulties of timely diagnosis:

“*The downside is that I’m seeing a very small percentage of people that need to be seen*. *Because they’re not recognized*. *People think ‘oh they’re old’ or ‘they have dementia’ without even knowing if they have dementia*. *Or ‘oh they have been in intensive care*, *of course they are going to be confused*.*’ So outside of the geriatric medicine it’s quite challenging*.*” (P13)*

It appeared that there were conflicting processes for delirium assessment and that most identification of delirium for research purposes relied on clinicians’ identification of delirium, rather than researcher assessment. This was seen as problematic because participants felt they could not rely on the accuracy of clinicians’ recognition and assessment of delirium:

“*The first is how to classify patients having delirium or not*. *Because we have to define whether the patient has delirium and sometimes when we are assessing the patient*, *he has no delirium*, *but we have previous reports from the nursing staff or from clinical records that the day before he was on delirium*. *So it’s difficult to classify this type of patient*.*” (P10)*

Participants readily acknowledged the difficulty of precisely defining delirium, noting that it is a syndrome that varies from person to person:

“*Because delirium is a set of signs and symptoms and it’s not necessarily a diagnosis that you make with histopathology or with very specific lab tests*. *So you may not detect delirium until a certain time point but that doesn’t mean the brain wasn’t injured prior to that time point*, *so there is a lot of uncertainty about when delirium started and when it’s resolved–these make it very challenging*.*” (P12)*

Others highlighted uncertainties with the classification of sub-syndromal delirium, noting that these individuals are often placed in the ‘control group’ (i.e. no delirium) in delirium biomarker studies:

“*I think when you use the binary of delirium–the yes/no it is because there can be symptoms present- like sub-syndromal delirium–and they’re not going to sell it by the full-blown delirium*. *[…] I think understanding the symptom burden at the time of the biomarker being drawn is really important […] maybe they are fluctuating and have some disorganised thinking but they don’t have inattention—so technically they can’t qualify as having delirium but some can certainly argue that there definitely is some brain dysfunction going on*. *Therefore*, *if they do not have a proper diagnosis of delirium at the time of blood draw then they would be categorised as non-delirious*. *So it’s introducing a lot of noise into the data*.*” (P07)*

#### 1b. Delirium superimposed on dementia (DSD)

DSD was a significant challenge mentioned by several participants, and the importance of adjusting for dementia in all delirium biomarker studies was highlighted:

“*If you are doing biomarker studies in delirium you really need to have a picture of the dementia status of the patient both because dementia is the strongest risk factor for delirium and because dementia also impacts on the biomarkers that you want to measure and sometimes the relation is in the opposite direction […] So if you don’t adjust for dementia in your analysis then they will level one another out*.*” (P11)*

The need to have multiple control groups in delirium biomarker studies to understand which biomarkers are affected by dementia was identified:

“*Well that’s why we are doing this study…to distinguish*. *We are classifying patients into four groups*. *So we have patients who are totally normal*, *with no delirium and no dementia*. *And then we have patients with dementia and delirium*, *then dementia without delirium and also patients with no dementia and [with] delirium*. *So we can compare the effects of delirium superimposed on dementia*.*” (P10)*

#### 1c. Hypothesis driven

The importance of taking into consideration the underlying biology of delirium by testing for a hypothesis was discussed. It was noted that *“there isn’t any thought going into it” (P15)* including about which biomarkers were being studied and why:

“*People are doing these studies with no eye on the biology*. *I mean I find it really frustrating […] Everyone is going–‘Ok we will just get this kit*, *put the 27 chemokines or cytokines on there*, *bang them on’*, *but there isn’t any thought going into it*. *For me*, *it’s a huge problem because no one is actually testing a hypothesis*. *I think that not enough biomarker studies have a real clear guiding principle*, *and that is a hypothesis that they are testing*. *Because if you are testing a hypothesis then you have to think about what it would take to provide support to the hypothesis*, *or to refute the hypothesis*. *I just feel that no one states a clear hypothesis*, *no one is studying a hypothesis so we just have very weak associations*.*” (P15)*

One participant noted that authors often concluded that there was a ‘dysregulation’ in inflammatory markers, without taking into account any *priori* hypothesis. The need to clearly state and define a hypothesis was perceived as one reason for weak associations in delirium biomarker studies:

“*And it means that if they do a panel of 27 markers and only 2 of them change*, *then they can just say ‘this provides evidence for inflammatory dysregulation in delirium’–and that’s of no value whatsoever*, *because if you look at 27 things then statistically at least one of them will change by chance*! *And therefore you are going to find something and if it goes up or down and you don’t really care which*, *because you can say ‘dysregulation’ either way and that means you’re going into a paper with zero hypothesis*, *you’re just saying throw it at the wall […] I find it very infuriating- those studies are not contributing to the knowledge of delirium*.*” (P15)*

#### 1d. Limited infrastructure and resource investment

The difficulties of conducting biomarker research without appropriate infrastructure was perceived as a potential barrier to rigorous delirium biomarker research:

“*I guess it’s difficult to do collection of samples for biomarker research or any kind when you don’t have the infrastructure*. *We have only just got a minus 80 freezer so basically if you were in a place that is not an academic centre and they haven’t given you a shelf for research samples that can be tricky […] It’s not impossible but it’s obviously useful to do research outside of academic*.*” (P6)*

Whereas another participant believed that there are fundamental principles of conducting and reporting delirium biomarker studies that should be adhered to if the results are to inform the field, regardless of funding.

“*I guess it’s a resource argument*. *But I disagree*, *because if we aren’t following some sort of guidelines then we are really doing our patients a disservice because we are not going to make any progress […] Whenever you draw a biomarker you should follow the same steps regardless of whether you have funding or not*. *You’re not saying what assay they should use*, *you’re saying when you write up your findings you need to share which assay and how they did it*. *I don’t see how you need money for that*.*” (P07)*

#### 1e. Fluctuating nature of delirium means time point of biomarker collection is a crucial consideration

Several participants acknowledged the great challenge with ensuring the right timing of biomarker collection due to the fluctuating nature of delirium:

“*They’re difficult*. *Essentially because delirium is normally fixed pretty quickly around the hospital environment*, *especially around geriatrics*. *There is a small window of finding those patients*.*” (P01)*

Some highlighted the need for longitudinal samples to track delirium over time:

“*And then you need to follow the patient*, *ideally several times a day to be safe*. *Because delirium episodes can be for maybe some hours*, *and it can develop during the weekend or during the night and if you don’t have a plan for how you are going to assess this information then you will lose it and falsely classify the patient as non-delirious*.*” (P11)*

However, other participants thought that longitudinal sampling was not always feasible:

“*You need to make a system where you still are able to pick up the CSF the day it comes and that is very hard unless you want to employ a person to be at the hospital 24/7—it will be extremely expensive*.*” (P11)*

#### 1f. Collecting CSF and imaging in people with delirium

CSF was considered the ‘gold standard’ in delirium biomarker research, due to the proximity to the brain, providing an advantage over blood. Despite most participants believing that CSF collection posed too many practical challenges, others emphasised the need for more CSF sampling, noting that it was more likely to directly reflect brain processes during delirium:

“*So the first problem is*, *in my opinion*, *you really need CSF*. *You cannot do delirium biomarker studies in blood*. *Well you can*, *but there are not so many good candidates for biomarkers in blood that give you good information about the brain*.*” (P11)*

Yet most participants spoke about the difficulties of CSF collection via lumbar puncture, namely its invasiveness and burden on patients:

“*CSF is not easy to get hold of because you need to do a lumbar puncture which is considered invasive*.*” (P11)*

Similarly, despite the great opportunity that neuroimaging has to offer, several participants focused on the practical challenges of imaging studies and the difficulties associated with undertaking a PET scan when a patient is agitated:

“*Yes well you can’t do a PET during the delirium*, *you would have to wait for the delirium to be resolved so that you can coach him through a PET session*.*” (P03)*

For this reason, there was a perceived bias towards hypoactive subtypes in PET studies, resulting in unrepresentative samples:

“*Yes that’s part of the other problems*. *We tend to have much more of a bias for the hypoactive delirium [in imaging studies]*.*” (P01)*

#### 1g. Accounting for the complexity/biology of the person as a whole

Majority of participants commented on the need to create a homogenous and “*clean*” cohort, acknowledging that people with delirium, particularly in the ICU, often had several underlying conditions affecting the results:

“*I think you want to have a really clean cohort and not too many comorbidities so if you want to come up with a biomarker that you want to associate with the disease process […] we need cleaner cohorts so we can isolate a biomarker that is specific to delirium*.*” (P09)*

In contrast, other participants concurred that the next step to broaden delirium biomarker studies is to biomarkers across several settings:

*“Well repeating it in more ICU patients might not be that helpful*. *For instance, it’s a lot easier for me to do it in the ICU because that’s where a lot of my research lies. If we really find something that hits then you—start looking at that biomarker in other populations. And if it’s hitting across multiple [populations] then that gives you a lot more confidence that it’s actually specific to delirium, right?” (P02)*

One participant argued that *“existing brain state is going to be the key determinant of whether those acute changes are enough to trigger delirium” (P15)*, therefore emphasising the need to obtain true baseline measurements. Not having a precise baseline was considered a major shortcoming in delirium biomarker studies:

“*I think a key practical challenge with delirium is that we don’t have baselines […] that’s particularly important for somebody with my mindset because I think your brain state before delirium is the major predictor of who will get delirium and how badly they will be affected*. *So the severity of the acute insult is obviously a major determinant*, *but who is vulnerable to having delirium in those situations—we learn about that by having a baseline*.*” (P15)*

The surgical space was considered the best setting for conducting delirium biomarker research with respect to having true baseline measurements:

“*I would say the best cohort is probably peri-operative and post-operative because you know exactly what kind of injury is happening and when it is happening and you can have a biomarker before the injury and then you can have the biomarker after the insult*.*” (P09)*

Some participants asserted that patients in this setting generally had less co-existing conditions that can influence the results and therefore can provide a more accurate depiction of the specific biomarkers for delirium:

“*You should need to take patients perhaps in surgery*. *So the hip fracture patient group is a possible patient group because they break their hips and you can distinguish these biomarkers that come from the hip fracture and those that come from the delirium so this is a very interesting population*. *Normally you don’t have sepsis*. *Normally you don’t have cancer or something like that*.*” (P08)*

On the other hand, others emphasised that the prevalence of delirium in this group was much lower, which subsequently introduces a selection bias:

“*If you do cognitive studies in elective surgery patients you will always have a selection bias*. *So if we look at the patients who participate in our studies they are cognitive at baseline*, *pre operatively*, *they are much better…three points lower …than if you take a random sample of the patients we treat here and that puts you in an awkward position*. *So there is a methodological flaw right from the start*.*” (P03)*

The heterogeneity of delirium causation was considered a major challenge which varied from person to person. The common approach of relying on clinical identification of delirium left people uncertain:

“*Delirium is so multifactorial so if you take an ICU patient*, *you have so many possible pathophysiological mechanisms that will lead to delirium […] That’s why it’s so heterogeneous and why it will never have a magic bullet or an overall approach to the problem*. *It’s different in every patient*. *In every patient*, *it’s his personal mix of mechanisms to go into delirium*. *That makes therapy so difficult because there are so many underlying causes […] so there are several mechanisms that lead to delirium that makes standardisation in studies nearly impossible*.*” (P03)*

When asked about accounting for underlying conditions present in people with delirium, participants acknowledged that, as a whole, delirium researchers have thus far inadequately tackled this issue:

“*Nobody is doing it [accounting for underlying conditions] and nobody knows what to do about it so it’s really good you are writing this*. *It will give some ideas to people*.*” (P09)*

#### 1h. Standardisation of delirium biomarker research

Participants reflected on the quality of current delirium biomarker research and highlighted the issue of poorly reported and/or conducted delirium biomarker studies:

“*We don’t do a very good job on the side of reporting and reporting that precision so it’s rather messy and a lot of the time unable to tell whether the person doing the biomarkers whether they were drawn before or during the delirium*.*” (P07)*

Participants asserted the need for reporting guidelines, highlighting that often researchers merely replicated procedures of others in the field without considering best practice methods:

“*I think our field is missing a metric or a standard to follow*. *So you just end up doing what your institution or other studies typically do and that’s how you report it*.*” (P07)*

Using the same protocols for assay procedures was considered important for standardisation, as well as for the potential to combine samples for larger delirium biomarker studies:

“*We should try to use similar protocols at different centres so it’s possible to combine samples […] You can also standardise the way you handle your samples after you collect them–just basic things like using the same tubes because some biomarkers that you want to analyse they can adhere… if you don’t use the correct material to collect the CSF then the proteins can adhere to the surface then you can’t trust your results*.*” (P11)*

### Valuing delirium research through investment and collaboration

#### 2a. Ethics committee barriers

Many participants shared a frustration towards ethics committees’ restrictions in relation to delirium biomarker studies, highlighting it as a notable barrier to progressing the field:

“*We are very restrictive for supporting this kind of research*. *For example*, *you won’t get patients with a very severe dementia and delirium because most of the ethical committees won’t let family members give proxy consent*.*’ (P08)*

A reason for the strict restrictions was the perception of ethics committees that patients did not directly profit from being involved in a delirium biomarker study:

*“We have a general problem with perception of doing research on patients*. *They think we use them like guinea pigs. Particularly with delirium research where you don’t have a personal profit. It is different if you are in the oncology and you are coming up with a treatment regimen—there you have a potential profit for yourself. In delirium research you don’t and they are very reluctant to say yes and go along with that.” (P03)*

There was a perception that ethics committees considered people with delirium too vulnerable to be included in research; hence, introducing a selection bias whereby cohorts in these studies often consisted of people with lower risk of delirium:

“*Essentially our ethics committees are getting more difficult*. *Many patients who have a high risk of delirium are a cognitively impaired at baseline so they fall into the category of vulnerable group of patients which makes it difficult to approach them*. *Then we have the problem that the … if you approach*, *you will get the good ones with too low rates of delirium*.*” (P03)*

A pragmatic solution to this barrier was to append the biomarker study onto an already existing trial, alleviating the hurdles of obtaining ethical approval for delirium biomarker studies:

“*Linking to some sort of ongoing trial that is enrolling people for another reason […] So I think linking on to randomised controlled trials or big observational cohorts*, *whatever they’re doing*, *getting funding and adding it on something that is co-existing is a lot easier*.*” (R02)*

In contrast, one participant took a long-term approach, and disagreed with tagging the biomarker component onto an existing study. They argued that in order to conduct robust delirium biomarker research, the studies must be “*bespoke*” and original:

“*If you want to do a really good biomarker study*, *or really good pathophysiology work then sometimes you just can’t build that on the back of routine clinical care*. *They have to be bespoke studies where you have to go the extra mile […] You have to write up a protocol that’s more involved*, *that asks more of the patient and carers […] It’s one of those things*, *that if you really want to advance the research*, *then you need to do a real research study*. *And by real*, *I mean bespoke*. *That’s not being critical of the opportunistic studies*, *but sometimes if you want to answer the hard questions*, *you have to do the hard studies*.*” (P15)*

#### 2b. Transdisciplinary collaboration

Participants described a number of areas where current delirium biomarker studies were falling short. They acknowledged that current studies were predominantly conducted by clinicians:

“*I think delirium is a relatively young field and it’s been driven primarily by clinicians which is great because they’re really invested or embedded in the health system next to the patient so you have that really rich clinical representation*. *But the down side is that they just aren’t necessarily trained very strong methodologically*.*” (P07)*

The importance of collaboration between clinicians and scientists to improve the science of delirium biomarker studies was highlighted by most:

“*I am not sure whether the basic scientists work on this topic*. *It’s more that delirium clinicians work on this type of research […] I think it’s about integrating these people into the study*.*” (P08)*

## Discussion

This study of delirium researchers’ perspectives about the key methodological challenges in the conduct and reporting of delirium biomarker research sheds light on the current state of the scientific field. Findings identified a range of factors that contribute to the challenges of conducting delirium biomarker research and the risk of the field not accelerating efforts, which have not previously been explicitly acknowledged or reported. It provides the most in-depth exploration of these challenges to date, and some important insights into how to address the many practical, scientific and quality issues in research into delirium pathophysiology.

### Practical and scientific challenges of delirium biomarker research: Stagnation versus driving improved methods and reporting

Overall, researchers in this study concurred that delirium biomarker research is in practical terms an extremely difficult and complex field. A minority took a long-term view, whereas many reported taking short-term approaches, even as they acknowledged that the latter was unlikely to advance scientific knowledge of delirium. Although the practical difficulties and complexities of delirium biomarker research was a common finding, some participants also provided clues and suggestions as to how some issues may be addressed.

The issue of delirium under-recognition and misdiagnosis by clinicians, which has been extensively studied and reported as occurring in 21% - 79% of cases across settings [18–20]. It appears from the present study that reliance on clinical identification of delirium, as opposed to researcher assessment, has contributed to much uncertainty about whether delirium was indeed present, or not, at the time of biomarker collection. This finding flags the urgent need for more systematic and reliable processes for delirium identification in research into its biomarkers, which will require greater involvement of researchers and reporting of diagnostic quality. Furthermore, there are conflicting methods in how the features of delirium are assessed for research purposes. The difficulties with classifying delirium sub-types was also highlighted. The ability to distinguish between the different etiologic subtypes will be critical to elucidate delirium pathophysiology and to develop effective treatments.

There was congruence in the researchers’ views that accounting for co-existing conditions in delirium was important but extremely challenging, and divergent views about how to resolve the question. Most were uncertain about how to tackle this topic, and yet addressing this uncertainty in a united way is crucial to advancing the field of research. Delirium superimposed on dementia (DSD) was considered a key challenge by researchers, who noted the importance of adjusting for dementia in delirium biomarker studies. Delirium is a risk factor for dementia, and is associated with worsening severity in individuals with existing dementia [21]. The prevalence of DSD in community and hospitalised settings is well documented and ranges between 22% and 89% in people aged 65 and older [22]. When dementia and delirium co-exist, it is difficult to ascertain whether the observed changes in a particular biomarker were related to the delirium, or confounded by the underlying dementia [23]. Animal models of delirium during dementia have been developed, which suggest that prior synaptic loss and microglial priming are predisposing factors for acute cognitive impairment induced by systemic inflammation [24]. Although this model is highly promising, further validation in more studies is required. There is also an urgent need to characterise these two conditions biologically and clinically in human studies. Including multiple control/comparator groups would help to elucidate the distinctions.

A challenge identified in this study was the acuity, fluctuating course and often brief duration of delirium. These factors make precise determination of its onset and resolution extremely difficult; and yet research recruitment and precision in the timing of biomarker collection is crucial in delirium biomarker studies to accurately capture the delirium episode [25]. Furthermore, pathophysiological processes may differ in active delirium compared to those individuals who are not yet delirious. A standardised way of determining delirium resolution is also required, as there is currently no consensus on the definition of delirium resolution [26].

The proximity of CSF to the brain makes it a good target for studying the pathophysiology of central nervous system conditions. Obtaining CSF for research purposes however has numerous practical challenges. Most delirium researchers discussed the burden of CSF collection by lumbar puncture (LP), and referred to the procedure as “invasive”. Although there is no literature on the experience of adults undergoing LP, there has been much research in children and adolescents. One study demonstrated that 75% of parents/caregivers of children who were scheduled to undergo an LP did not consent because of the fear of complications [27]. One proposed solution to this barrier is to improve the quality and person-centeredness of information given to potential participants, to increase their understanding of the proposed research. A recent scoping review reported that many older people were willing to participate in research in the event of reduced decision-making capacity from a desire to contribute to scientific knowledge, although less so in studies with higher risks or burdens for them [28]. Reducing study risks and burdens, as well as improved communication processes with potential participants and proxies, are therefore crucial. For example, simplified information and consent forms using lay language that avoids medical jargon as well as extended discussions can lead to improvements in participant understanding and appreciation of study information [29, 30].

Neuroimaging is another method that has sparked interest in attempts to understand the neural correlates of delirium. Neuroimaging is routinely used in clinical practice; however, there are still very few studies on neuroimaging in delirium, which likely reflects the practical and ethical challenges involved in imaging patients with hyperactive delirium. Delirium researchers in this study expressed concerns about the practical challenges of getting a person who is agitated to lie still in a PET scanner. One solution is to ensure patients are accompanied by a relative or carer to reassure them prior to and during the scans, as was effectively enacted in another study [31]. Although imaging studies are deemed to be extremely difficult, large samples which adjust for confounding factors (for example, pre-existing cognitive impairment) are needed, as well as long-term vision and planning of research programs to facilitate the advancement of adequately powered studies [32].

The need to account for and understand the complexity and biology of the whole person was highlighted as a gap in current delirium biomarker studies. A key limitation of many previous studies in acutely admitted patients was the lack of objective cognitive testing at baseline, therefore making it difficult to know if any observed changes in biomarkers were related to the delirium, or were confounded by underlying conditions. Many researchers suggested that future delirium biomarker studies focus on the surgical setting, where patients have a true pre-operative baseline. The limitation of this approach is that delirium is a multifactorial condition, which almost always occurs in the context of other physiological processes that need to be accounted for in study participants.

This study confirmed that standardised methods in the form of reporting guidelines for delirium biomarker research are urgently required, as was initially identified in a previous systematic review [11]. Inadequate and/or unclear reporting of methodological processes can lead to discrepancies in results, which may be misleading and potentially detrimental to the research [33]. Overall, reporting guidelines are deemed necessary to promote studies that are standardised and reliable. This statement is consistent with other studies that reported improvements in reporting rigor when reporting guidelines such as the CONSORT (Consolidated Standards of Reporting Trials) [34] were adopted. Many journals have taken steps to improve the quality of the research articles that they publish by requiring the use of reporting guidelines, although research shows there is still room for improvement [35]. Having global standardised guidelines to conduct delirium biomarker research with similar reference standards will help to improve the quality of reporting within studies and thereby increase opportunities for syntheses across studies.

### Valuing delirium research through investment and collaboration

There are several ethical challenges to conducting research in patient populations at higher risk of harm, such as delirious patients who are often considered too vulnerable for research participation [36]. There is an ethical tension in delirium research; balancing the need to protect this more vulnerable population with upholding their rights to be included in research and the need to improve medical care [25]. This study confirmed that ethics committee interpretation of current research regulations when applied to delirium research are perhaps exceedingly stringent. This is driven by several factors: patients are unlikely to directly profit from participating in a delirium biomarker study, concerns about potential harms to a vulnerable population, perceived burden of specimen collection and the quality of informed consent. Those with impaired capacity are often either excluded from research or less frequently recruited, to circumvent the challenges of tailoring methods and study measures [28]. However, this evasion leads to unrepresentative study populations and thereby limits external validity of the research [25, 37].

Common motivations of older people to participate in research in the context of impaired decision-making include altruism, potential personal benefits, and a desire to contribute to scientific knowledge [28]. Greater consumer (e.g. people who have previously experienced delirium or their caregivers) input into delirium biomarker study development would help to ensure improved value proposition and communication by researchers to ethics committees and potential participants/proxies so they can better weigh the rewards/risks of delirium studies might help to overcome some of the barriers identified in this study.

The common approach of relying on the clinical identification of delirium within biomarker research should be replaced with a more rigorous process. Such a process could be elucidated by clinicians, scientists and researchers working in a more united way to improve methods in delirium biomarker research. This issue was identified in this study by the frequent acknowledgement that currently delirium biomarker research is predominantly conducted by clinicians with minimal background in basic science. To address these gaps, multi-institutional collaborative efforts are needed to generate valid, reproducible and generalisable findings in delirium biomarker research. The Successful Aging after Elective Surgery (SAGES) [32] program is one example of a collaborative project aiming to achieve research rigour and results that would likely be unattainable by investigators working independently.

### Implications for research

Delirium is a major clinical and public health concern, and robust scientific research on pathophysiological mechanisms are urgently needed. Developing reporting guidelines is an essential step to improving methodological and reporting quality in delirium biomarker research. Increased international, multisite and transdisciplinary collaboration, along with concept development workshops focused on methodology of conducing delirium biomarker research at international delirium society meetings, would enable improvements in the field. Furthermore, better explanation of study rationales to ethics committees, and involvement of consumers, could help in alleviating some of the challenges identified in this study. Despite many studies seeking to better understand the pathophysiology of delirium, these barriers continue to impede high-quality delirium biomarker research. Raising awareness and changing practice and culture offer the multidimensional effort that is needed to progress this fundamental field of delirium research. Details regarding our recommendations for future research are given in Table 2.

**Table 2:** Recommendations for future research.

Interview theme	Recommendation
Practical and scientific challenges of delirium biomarker research: stagnation versus driving improved methods and reporting	
*Accuracy of diagnostic assessment of delirium*	Development of a reference standard for the diagnosis of delirium is needed.
*Delirium superimposed on dementia (DSD)*	In acutely admitted patients, assessments on cognitive decline should be used to assess dementia status. The use of multiple control/comparator groups could help elucidate the distinctions.
*Hypothesis driven *	Pre-defined hypotheses need to be supported by a strong biological underpinning.
*Limited infrastructure and resource investment*	Standardising protocols to allow for future collaborations between laboratories is essential.
*Fluctuating nature of delirium means time point of biomarker collection is a crucial consideration*	A standardised way of determining delirium resolution is required.
*Collecting CSF and imaging in people with delirium*	Person-centeredness is essential to increase participants understanding of the proposed research.
*Accounting for the complexity/biology of the whole person*	In elective studies, patients should undergo objective cognitive testing to obtain a true baseline before biomarker sampling.
*Standardise delirium biomarker research*	Reporting guidelines specific to delirium biomarker studies are needed.
Valuing delirium research through investment and collaboration	
*Ethics committee barriers*	Greater consumer input into delirium biomarker study development would help to ensure improved value proposition and communication by researchers to ethical committees and potential participants.
*Transdisciplinary collaboration*	Ongoing international, multisite and transdisciplinary collaboration, including concept development workshops on delirium biomarker research is essential.

### Strengths and limitations

A key strength of this study was the inclusion of participants from multiple disciplines and countries who were actively involved in delirium research, allowing data saturation to be reached. Secondly, the qualitative method allowed for an in-depth exploration into the reasons underpinning the participant views, giving clearer guidance of the specific areas for advancement in the field.

Participants were purposefully sampled in order to facilitate in-depth exploration delirium researchers’ perspectives, and so these findings are likely to be specific to the challenges of delirium biomarker research, rather than be transferable to biomarker research more generally. We are unsure if the predominance of male and clinician researcher participants is representative of the field, or had any particular influence on the findings of the study; however, this is worth noting as a potential limitation. Another limitation was that almost all participants in the study were from high-income countries.

## Conclusion

Findings of this qualitative study identified a range of factors that contribute to the challenges of conducting delirium biomarker research, which have not previously been explicitly acknowledged or reported. These factors all contribute to the overall quality of research in this field. Findings complemented the preceding systematic review and Delphi survey, and together these studies will inform strategies to improve the methods and reporting of delirium biomarker research. A concerted effort is now required to standardise and strengthen several aspects of the conduct and reporting of delirium biomarker studies, in order to advance this highly promising but yet to deliver scientific field of research.
